# Telehealth Expansion and Medicare Beneficiaries’ Care Quality and Access

**DOI:** 10.1001/jamanetworkopen.2024.11006

**Published:** 2024-05-13

**Authors:** Morteza Saharkhiz, Tanvi Rao, Sara Parker-Lue, Sara Borelli, Karin Johnson, Guido Cataife

**Affiliations:** 1 Meta Platforms, Inc, Menlo Park, California; 2 American Institutes for Research, Arlington, Virginia

## Abstract

**Importance:**

Understanding the association of telehealth use with health care outcomes is fundamental to determining whether telehealth waivers implemented during the COVID-19 public health emergency should be made permanent. The current literature has yielded inconclusive findings owing to its focus on select states, practices, or health care systems.

**Objective:**

To estimate the association of telehealth use with outcomes for all Medicare fee-for-service (FFS) beneficiaries by comparing hospital service areas (HSAs) with different levels of telehealth use.

**Design, Setting, and Participants:**

This US population-based, retrospective cohort study was conducted from July 2022 to April 2023. Participants included Medicare claims of beneficiaries attributed to HSAs with FFS enrollment in Parts A and B.

**Exposures:**

Low, medium, or high tercile of telehealth use created by ranking HSAs according to the number of telehealth visits per 1000 beneficiaries.

**Main Outcomes and Measures:**

The primary outcomes were quality (ambulatory care–sensitive [ACS] hospitalizations and emergency department [ED] visits per 1000 FFS beneficiaries), access to care (clinician encounters per FFS beneficiary), and cost (total cost of care for Part A and/or B services per FFS Medicare beneficiary) determined with a difference-in-difference analysis.

**Results:**

In this cohort study of claims from approximately 30 million Medicare beneficiaries (mean [SD] age in 2019, 71.04 [1.67] years; mean [SD] percentage female in 2019, 53.83% [2.14%]) within 3436 HSAs, between the second half of 2019 and the second half of 2021, mean ACS hospitalizations and ED visits declined sharply, mean clinician encounters per beneficiary declined slightly, and mean total cost of care per beneficiary per semester increased slightly. Compared with the low group, the high group had more ACS hospitalizations (1.63 additional hospitalizations per 1000 beneficiaries; 95% CI, 1.03-2.22 hospitalizations), more clinician encounters (0.30 additional encounters per beneficiary per semester; 95% CI, 0.23-0.38 encounters), and higher total cost of care ($164.99 higher cost per beneficiary per semester; 95% CI, $101.03-$228.96). There was no statistically significant difference in ACS ED visits between the low and high groups.

**Conclusions and Relevance:**

In this cohort study of Medicare beneficiaries across all 3436 HSAs, high levels of telehealth use were associated with more clinician encounters, more ACS hospitalizations, and higher total health care costs. COVID-19 cases were still high during the period of study, which suggests that these findings partially reflect a higher capacity for providing health services in HSAs with higher telehealth intensity than other HSAs.

## Key Points

**Question:**

Was telehealth use associated with quality, access, and cost of care outcomes for fee-for-service Medicare beneficiaries during the COVID-19 telehealth expansion?

**Findings:**

This cohort study compared 3436 hospital service areas (including approximately 30 million Medicare beneficiaries) with different levels of telehealth use in a difference-in-differences analysis framework. Compared with areas with low telehealth use, high telehealth use was associated with more ambulatory care–sensitive (ACS) hospitalizations (1.63 additional hospitalizations per 1000 beneficiaries per semester), no additional emergency department visits, more clinician encounters (0.30 additional clinician encounters per beneficiary per semester), and higher total cost of care ($164.99 higher cost per beneficiary per semester).

**Meaning:**

Data from the COVID-19 pandemic suggest that higher levels of telehealth use may be associated with increased access to care and potentially lower quality of care (evidenced by increases in ACS hospitalizations); replication of this analysis with post–COVID-19 data is needed to fully understand the impacts of the telehealth expansion.

## Teaser

This cohort study examines the association of telehealth use during the COVID-19 telehealth expansion with hospitalizations, emergency department visits, clinician encounters, and costs among Medicare fee-for-service beneficiaries.

## Introduction

Medicare’s expansion of coverage for telehealth during the COVID-19 public health emergency (PHE)^1^ led to sharply increased use of these services.^2,3^ Telehealth visits for Medicare fee-for-service (FFS) beneficiaries increased 63-fold in 2020, from approximately 840 000 visits in 2019 to nearly 52.7 million visits in 2020.^4^ Telehealth use peaked in the second quarter of 2020 (47% of Medicare users with a telehealth service) and leveled off by the end of 2022 (15%).^5^ Nevertheless, compared with prepandemic levels, telehealth use remains remarkably high. Even though the PHE ended in May 2023, Congress extended many Medicare telehealth expansions through December 2024 to assess telehealth’s impact on health care outcomes more thoroughly and to inform potential permanent changes to telehealth coverage policy.^6^

The current literature on the association of telehealth use with outcomes such as health care utilization and quality is small and has yielded mixed results. Depending on which health care system was analyzed, telehealth has been found to be associated with both no increase in utilization (with telehealth substituting for in-person visits)^7^ and increased primary care utilization.^8^ Similarly, some studies have found telehealth use to be associated with lower health care quality, whereas others have found the opposite. A study^9^ using data from 1 urban integrated academic health system in California found that telehealth encounters were associated with higher repeat hospitalizations and emergency department (ED) visits than in-person encounters, and another study^10^ using data from a single commercial payer found that telehealth visits require more follow-up for acute conditions than in-person encounters. However, a recent study^11^ of Medicare patients aged 65 years or older from an accountable care organization in the Midwest found no significant difference between telehealth and in-person encounters in terms of the number of days until the next visit or the probabilities of 3-day and 7-day follow-up visits. Another study^12^ documented a largely favorable association between telehealth exposure and the quality of primary care using electronic medical record data of commercial, Medicare, and Medicaid beneficiaries from more than 200 outpatient care sites in Pennsylvania and Maryland. Differences in telehealth protocols across health care systems may have contributed to the inconclusive findings.^13^ In addition, most published studies compare telehealth users with nonusers, attempting to control for confounders but acknowledging the inherent bias. As an exception, Li et al^14^ categorized practice-level telehealth into terciles using Michigan commercial payer data; they found that patients from practices with higher telehealth use had a higher rate of ambulatory care–sensitive (ACS) hospitalizations and ED visits.

The objective of our study is to assess the association of telehealth expansion with health care outcomes among all Medicare FFS beneficiaries. This is an important contribution to the literature given the various methodological limitations of previous studies, which were generally small; focused on select states, practices, or health systems; and compared telehealth users with nonusers. We use a population-based difference-in-differences (DID) method to test whether the telehealth expansion is associated with improved quality and access to care, as well as lower health care costs. Some of the results of this analysis were included in the Medicare Payment Advisory Commission (MedPAC) June 2023 Report to Congress.^6^

## Methods

### Study Design, Setting, and Participants

In this cohort study, we used a DID design to estimate the association of telehealth use with quality of care, access to care, and cost of care for FFS Medicare beneficiaries. The baseline period is the second semester of 2019 (before the PHE and the expansion of telehealth), and the treatment period is the second semester of 2021 (after COVID-19 vaccines were widely available and the expansion of telehealth). The study population includes all Medicare FFS beneficiaries who had Part A and B enrollment during the entire semester and were alive as of the first day of that semester. Data are aggregated at the hospital service area (HSA) level, which is the unit of analysis. HSAs are local health care markets that satisfy most of the residents’ health care needs, including hospitalizations, and the outcomes we analyze are intended to assess quality and access provided by local health care systems.^15^ Aggregating to the HSA level also lessens concerns about selection bias from comparing telehealth users and nonusers. Beneficiaries are attributed to an HSA using the first zip code for that year and semester. Informed consent was not sought because the data used in this study do not involve human research participants, as defined in 45 CFR §46.102(e). Because the study did not use identifiable private data, nor was there any intervention or interaction with participants, the study was determined to be exempt from institutional review board oversight by the American Institute for Research’s institutional review board. This study conforms to the Strengthening the Reporting of Observational Studies in Epidemiology (STROBE) reporting guideline.^16^

### Variables

We created 3 levels of intensity for the usage of telehealth services, our exposure variable, by ranking HSAs according to the number of telehealth visits per 1000 beneficiaries in the second half of 2021 (telehealth was restricted before the PHE). The outcomes are quality of care, including ACS hospitalizations and ED visits per 1000 FFS Medicare beneficiaries; access to care, including clinician encounters per FFS Medicare beneficiary, with a breakdown by clinician type; and total cost of care for Part A and Part B services per FFS Medicare beneficiary with a breakdown of cost by service type. Clinician encounters are office visits identified using the Carrier claim file (excluding ambulatory surgery center services). Each encounter is a unique combination of beneficiary identification, claim identification, and National Provider Identifier.

We controlled for the following HSA Medicare population characteristics: share of Medicare beneficiaries enrolled in FFS Medicare; share of Medicare FFS beneficiaries by age, sex, race, and Medicaid eligibility; mean hierarchical condition category risk score (and its square); share of FFS beneficiaries attributed to alternative payment models; and mean Area Deprivation Index (ADI) for FFS Medicare beneficiaries. We also controlled for HSA market characteristics: population size and new (in a given semester) and cumulative (from the start of the PHE) COVID-19 cases per 10 000 people.

### Data Sources and Measurement

We identified telehealth visits using the Carrier physician and outpatient claims Standard Analytic Files. Outcome variables were calculated using FFS Medicare claims and the Common Medicare Environment custom enrollment file. Beneficiary covariates were calculated using the Common Medicare Environment custom enrollment file, Risk Adjustment System data, and Master Data Management beneficiary extract. We obtained the ADI from the University of Wisconsin School of Medicine and Public Health,^17^ population data from the US Census Bureau,^18,19,20^ and the number of COVID-19 cases from the *New York Times* database.^21^ For population size and COVID-19 cases, which are at the county level, we created a crosswalk between counties and HSAs. Data on race and ethnicity (Asian, Black, Hispanic, and non-Hispanic White, unknown race, and any other race not otherwise specified) were obtained from Medicare enrollment files and are included in this study along with other demographic characteristics to control for changes over time within geographic areas that could confound the findings.

### Statistical Analysis

The analyses were conducted from July 2022 to April 2023 using StataMP version 16.1 (StataCorp). The Figure illustrates whether trends were parallel across groups by visually comparing outcome pretrends for the 3 treatment groups for 2018 and 2019. We analyzed descriptive statistics of demographic characteristics, comparing the second semester of 2019 and 2021, as well as across the 3 groups in the second semester of 2019. We conducted a linear regression with fixed-effects DID analysis that identifies the association of telehealth intensity by comparing the mean change in an outcome between 2019 and 2021 for HSAs with medium or high telehealth intensity with the mean change in that outcome for HSAs with low telehealth intensity during the same period. We adjusted for time-varying confounders using the covariates described earlier for 2019 and 2021. We estimated heteroskedastic robust SEs and clustered them at the HSA level. Statistical significance was defined as a 2-sided *P* < .05.

**Figure.**
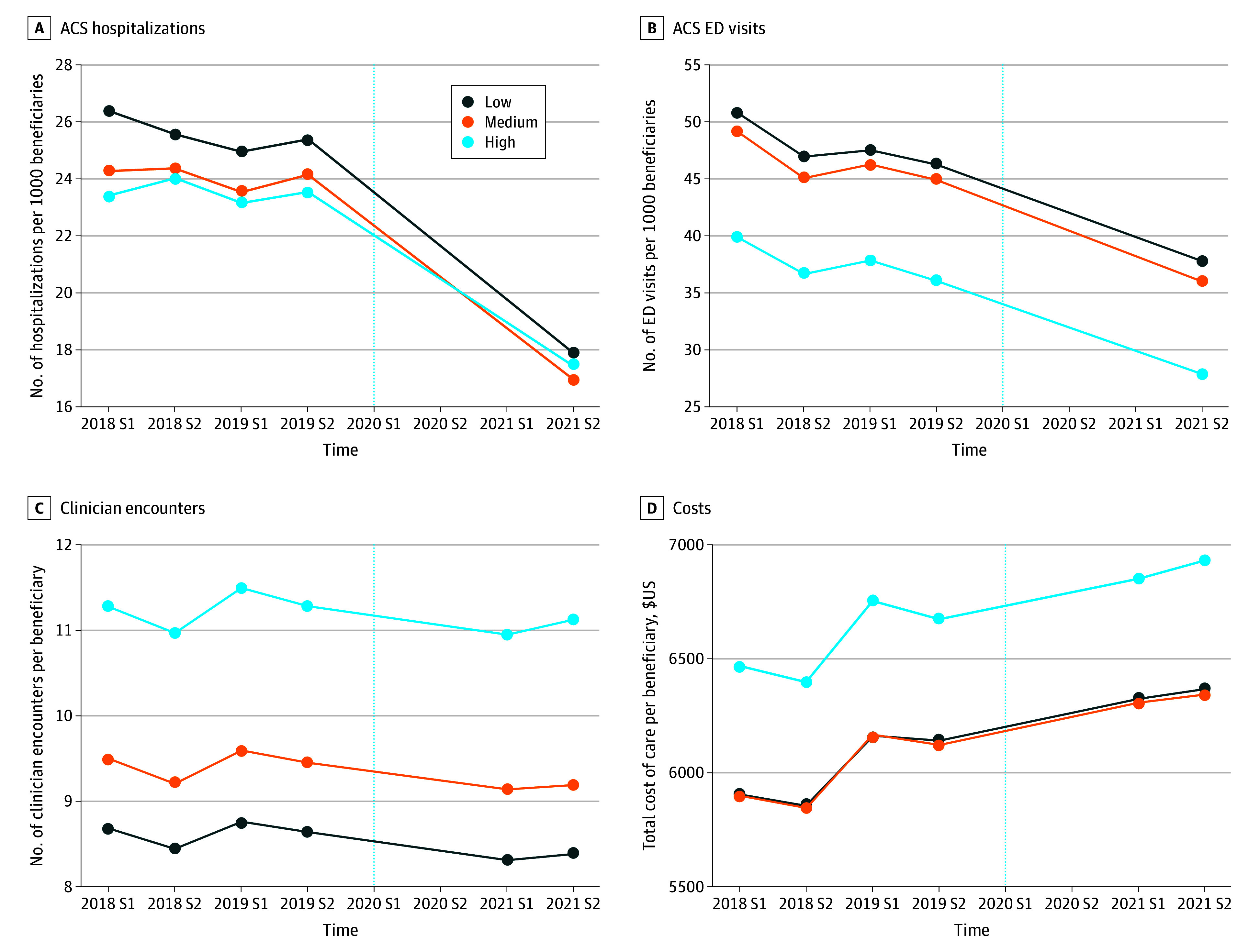
Outcome Measures by Telehealth Tercile Over Time The vertical dashed line shows the beginning of the public health emergency. ACS indicates ambulatory care sensitive; ED, emergency department; S, semester.

We conducted several sensitivity analyses. First, we controlled for geographic adjustment factors using the hospital wage index from the Inpatient Prospective Payment System and measures of geographic practice cost indexes, because Medicare payments change from year to year as a result of changes in geographic adjustment factors. Second, we controlled for in-person utilization because the association of telehealth usage may be confounded with that of general health care utilization. Third, we excluded small HSAs (with <500 beneficiaries) because ACS hospitalizations and ED visit rates calculated using a denominator with fewer than 500 beneficiaries have low reliability. Fourth, we used quartiles of telehealth usage to define treatment. Fifth, we ran separate estimates for rural and urban HSAs because the high telehealth intensity group has higher levels of urbanicity and because differences in outcomes could, thus, be due to differences in urbanicity levels rather than telehealth usage. In addition, we estimated a propensity score–weighted DID, where weights were obtained using multinomial logistic regression that estimated the probability of an HSA having low, medium, or high telehealth intensity on the basis of its observed characteristics during the second semester of 2019. Propensity score weighting makes low, medium, and high groups more comparable on observed characteristics and, presumably, on unobserved confounders as well.

## Results

### Sample Characteristics

In the 3436 HSAs analyzed, the sample declined from 31 625 338 beneficiaries in 2019 to 29 335 407 beneficiaries in 2021, mostly owing to increasing Medicare Advantage enrollment (mean [SD] age in 2019, 71.04 [1.67] years; mean [SD] percentage female in 2019, 53.83% [2.14%]). Nonetheless, the characteristics of beneficiaries in the sample were largely unchanged (Table 1).

**Table 1.** Sample Characteristics^a^

Characteristic (n = 3436 hospital service areas)	Mean (SD)	
	2019	2021
No. of beneficiaries alive and with Medicare fee-for-service Part A and B for 6 mo	31 625 338	29 335 407
Age, y	71.04 (1.67)	71.50 (1.51)
Sex, %		
Female	53.83 (2.14)	53.78 (2.16)
Male	46.17 (2.14)	46.22 (2.16)
Race and ethnicity, %		
Asian	1.52 (4.44)	1.58 (4.57)
Black	6.14 (11.06)	5.53 (10.12)
Hispanic	4.34 (9.11)	4.25 (8.86)
Non-Hispanic White	84.27 (16.77)	84.77 (16.23)
With full Medicaid eligibility for 6 mo, %	12.65 (7.58)	12.55 (8.16)
With partial Medicaid eligibility for 6 mo, %	5.75 (4.25)	4.64 (3.66)
Attributed to an Alternative Payment Model for at least 1 mo, %	34.76 (22.33)	34.10 (21.92)
Living in urban areas, %	47.37 (47.66)	47.38 (47.63)
Area Deprivation Index	61.41 (22.15)	60.93 (22.18)
Cumulative No. of COVID-19 cases/10 000 people	NA	1722.98 (366.73)
No. of new COVID-19 cases/10 000 people	NA	695.54 (193.43)

Abbreviation: NA, not applicable.

^a^ The statistics pertain to the second half of the year. Note that demographics are reported as means or percentages at the hospital service area level, and this table reports the averages of these means and percentages.

The mean telehealth visits per 1000 FFS Medicare beneficiaries were 174 visits in the low intensity group, 311 in the medium intensity group, and 679 in the high telehealth intensity groups. The terciles were similar in terms of age and gender, but urbanicity and the ADI differed across groups. The high group was also more ethnically diverse and included a higher number of low-income beneficiaries (Table 2).

**Table 2.** Demographic Characteristics by Telehealth Tercile at Baseline^a^

Characteristic	Mean (SD)		
	Low	Medium	High
Age, y	71.26 (1.53)	70.74 (1.52)	71.14 (1.89)
Sex, %			
Female	53.69 (1.98)	53.37 (2.02)	54.46 (2.27)
Male	46.31 (1.98)	46.63 (2.02)	45.54 (2.27)
Race and ethnicity			
Asian	0.35 (0.38)	0.63 (1.38)	3.61 (7.15)
Black	5.42 (11.50)	5.87 (10.35)	7.13 (11.26)
Hispanic	2.19 (5.97)	2.77 (5.88)	8.10 (12.62)
Non-Hispanic White	88.19 (14.94)	87.02 (13.53)	77.49 (19.30)
With full Medicaid eligibility for 6 mo, %	11.02 (5.18)	11.77 (5.68)	15.18 (10.21)
With partial Medicaid eligibility for 6 mo, %	6.24 (4.36)	6.27 (3.99)	4.71 (4.22)
Attributed to an Alternative Payment Model for at least 1 mo, %	33.24 (23.62)	34.32 (22.16)	36.73 (21.00)
Living in urban areas, %	24.39 (39.91)	41.15 (46.44)	76.77 (40.49)
Area Deprivation Index	72.98 (12.67)	66.24 (16.76)	44.86 (24.63)
No. of hospital beds/10 000 people	37.30 (45.41)	29.15 (28.74)	26.11 (19.57)
No. of primary care physicians/10 000 people	10.49 (5.29)	11.46 (5.57)	14.98 (6.31)

^a^ Low, medium, and high denote telehealth intensity groups in the second semester of 2021. All statistics are a mean over hospital service areas (HSAs) and pertain to the second semester of 2019. Note that demographics are reported as means or percentages at the HSA level, and the table reports the mean and variance of these means and percentages. Means by HSA over time can be found in eTable 14 in Supplement 2.

### Outcome Data

Between the second semester of 2019 and the second semester of 2021, mean ACS hospitalizations (−8.14 ACS hospitalizations per 1000 beneficiaries, 32.05% of the baseline rate) and ED visits (−12.12 ACS ED visits per 1000 beneficiaries per semester, 26.22% of the baseline rate) declined sharply, whereas the mean total cost of care per beneficiary per semester increased slightly ($424.54; 6.92% of the baseline rate). Mean clinician encounters per beneficiary declined slightly (−0.23 clinician encounters per beneficiary per semester; 2.72% of the baseline rate). The trends by tercile are shown in the Figure.

### Changes in Quality, Access, and Costs by Telehealth Intensity

#### Quality Outcomes

Table 3 presents the DID regression results adjusted for covariates. Without adjusting for covariates, the high group had 1.39 additional ACS hospitalizations per 1000 beneficiaries (95% CI, 0.84-1.94 hospitalizations) compared with the low group. This difference remained mostly unchanged (1.63 additional hospitalizations; 95% CI, 1.03-2.22 hospitalizations) when we controlled for covariates (Table 3). Given the decline in ACS hospitalizations (Figure), this implies that mean ACS hospitalizations decreased in the high group but at a slower rate than in the low group. There was no statistically significant difference in ACS ED visits between the low and high groups, or between the low and medium groups for ACS hospitalizations or ED visits (Table 3).

**Table 3.** Differences in Outcome Trends by Telehealth Tercile Groups (Difference in Differences Regression Results)^a^

Variable (n = 6872)	Difference vs low telehealth HSAs (95% CI)	*P* value	Percentage of base rate
ACS hospitalizations/1000 beneficiaries/semester			
Medium	0.41 (−0.21 to 1.03)	.19	1.70
High	1.63 (1.03 to 2.22)	<.001	6.90
ACS emergency department visits/1000 beneficiaries/semester			
Medium	−0.58 (−1.72 to 0.56)	.32	−1.29
High	0.10 (−0.87 to 1.06)	.84	0.27
Mean clinician encounters per beneficiary per semester			
Medium			
Total	0.02 (−0.04 to 0.09)	.51	0.22
Primary care	0.00 (−0.02 to 0.02)	.85	0.14
Specialists	−0.03 (−0.07 to 0.00)	.08	−0.62
APRNs and PAs	0.04 (0.02 to 0.05)	<.001	2.77
Other practitioners	0.01 (−0.00 to 0.03)	.10	0.94
Hospitalists	0.00 (−0.01 to 0.01)	.58	0.57
High			
Total	0.30 (0.23 to 0.38)	<.001	2.67
Primary care	0.04 (0.01 to 0.06)	.006	1.95
Specialists	0.09 (0.05 to 0.13)	<.001	1.49
APRNs and PAs	0.08 (0.06 to 0.10)	<.001	6.60
Other practitioners	0.07 (0.05 to 0.09)	<.001	4.00
Hospitalists	0.03 (0.02 to 0.04)	<.001	7.11
Mean total cost of care per beneficiary per semester, $US			
Medium			
Total	18.08 (−42.47 to 78.63)	.56	0.30
Inpatient	5.66 (−22.75 to 34.06)	.70	0.33
Outpatient	−22.07 (−53.22 to 9.08)	.17	−1.19
Skilled nursing facility	25.90 (−7.20 to 59.01)	.13	5.94
Home health	−7.44 (−12.31 to −2.56)	.003	−3.75
Hospice	0.63 (−4.01 to 5.26)	.79	0.55
Physician	12.05 (−3.85 to 27.95)	.14	0.74
Durable medical equipment	3.35 (−0.02 to 6.73)	.05	1.87
High			
Total	164.99 (101.03 to 228.96)	<.001	2.47
Inpatient	63.63 (35.09 to 92.17)	<.001	3.22
Outpatient	−31.93 (−66.12 to 2.26)	.07	−1.98
Skilled nursing facility	45.14 (16.39 to 73.89)	.002	9.51
Home health	−20.47 (−26.96 to −13.98)	<.001	−7.60
Hospice	−1.60 (−6.78 to 3.57)	.54	−1.27
Physician	100.54 (80.76 to 120.33)	<.001	4.89
Durable medical equipment	9.68 (4.30 to 15.06)	<.001	6.12

Abbreviations: ACS, ambulatory care sensitive; APRN, advanced practice registered nurse; HSA, hospital service area; PA, physician assistant.

^a^ Low, medium, and high denote telehealth intensity. Estimates show the change between the second semester of 2019 and the second semester of 2021 using a regression-based difference-in-differences estimation with controls (see Methods section). The denominator for the percentages is that group’s mean in the second semester of 2019. Full regression results are available in eTables 1 to 7 in Supplement 2.

#### Access Outcomes

The unadjusted difference between the medium and low groups in mean total clinician encounters per beneficiary per semester was not statistically significantly different from 0 (0.00; 95% CI, −0.04 to 0.04). After adjusting for covariates, there was still no statistically significant difference between the medium and low groups in total clinician encounters (Table 3). However, the medium group had a 0.04 increase in encounters (95% CI, 0.02 to 0.05 encounters) with advanced practice registered nurses (APRNs) and physician assistants (PAs) compared with the low group. This was offset by a 0.03 decrease in encounters with specialists (95% CI, −0.07 to 0.00 encounters), which likely explains the absence of a statistically significant association for encounters with all types of clinicians.

Unadjusted, the high group had an additional 0.10 mean total clinician encounters per beneficiary per semester (95% CI, 0.06-0.14 encounters) than the low group. After controlling for covariates, the high group had an increase of 0.30 clinician encounters per beneficiary per semester (95% CI, 0.23-0.38 encounters), which is 2.67% of the baseline rate. Although the analysis shows that the largest increases in the level of clinician encounters occurred among specialists, for whom encounters increased by 0.09 per beneficiary (95% CI, 0.05-0.13 encounters), this was the smallest change relative to the baseline rate (1.49%). The largest increases for the high telehealth intensity HSAs come from hospitalist encounters, which increased by 0.03 (95% CI, 0.02-0.04 encounters; 7.11% of the baseline rate). Similarly, encounters with APRNs and PAs, primary care physicians, and other practitioners (including psychologists and social workers) all increased: 6.60% for APRNs and PAs and 4.00% for other practitioners (Table 3).

#### Cost Outcomes

There was no statistically significant difference in total cost of care per beneficiary per semester whether or not we adjusted for covariates (−$5.79; 95% CI, −$64.59 to $53.00), but there were 2 significant changes to specific categories of costs (adjusted for covariates) that approximately offset one another. Home health costs decreased compared with the low group by $7.44 (95% CI, −$12.31 to −$2.56), whereas durable medical equipment (DME) costs increased by $3.35 (95% CI, −$0.02 to $6.73). Although these estimates do not exactly offset one another, the resulting difference is not statistically significantly different from 0. The analysis shows no statistically significant difference between the medium and low groups for the cost of inpatient care, outpatient care, skilled nursing facility (SNF) care, hospice care, or physician services (Table 3).

Without adjusting for covariates, the difference in total cost of care between the high and low group was not statistically significantly different from 0 ($30.16; 95% CI, −$22.64 to $82.97). After adjustment, the high group showed a statistically significant increase ($164.99 overall; 95% CI, $101.03 to $228.96; 2.47% of baseline) in total cost of care per beneficiary per semester. There were statistically significant increases in SNF care, DME, physician services, and inpatient care, whereas costs decreased for home health and outpatient care (Table 3). The analysis showed no statistically significant association with hospice care.

### Sensitivity Analyses

The statistically significant results were robust to all sensitivity analyses described in the Methods section, with only marginal differences (see eAppendixes 1 and 2 in Supplement 1 and eTables 1-12 in Supplement 2). The parallel trends assumption is satisfied for most, but not all, outcomes and/or preperiods. Full details are shown in eAppendix 3 in Supplement 1 and eTable 13 in Supplement 2. Means by HSA over time can be found in eTable 14 in Supplement 2.

## Discussion

This cohort study compared quality, access, and costs between the second half of 2019 and second half of 2021 for HSAs with different levels of telehealth intensity using Medicare administrative data for all FFS beneficiaries. We found mixed results.

During the study period, there were large decreases in ACS hospitalizations overall, but less so in areas with high telehealth intensity compared with the low telehealth intensity areas. We found no association with ED visits. The finding for ACS hospitalizations necessitates a nuanced interpretation. In general, reduced ACS hospitalizations are considered evidence of improved quality; however, during the PHE, less reduction may have been a marker of hospital capacity. Our measures of ACS hospitalizations and ED visits may have been directly affected by the PHE. One study^22^ found that respiratory-related ACS hospitalizations declined substantially starting early in the pandemic (around March 2020) and continuing until the end of that study (March 2021), potentially confounding our results.

We found that HSAs with high telehealth intensity had increases in clinician encounters for all clinician types compared with the low telehealth intensity areas. Telehealth may have increased accessibility by enabling visits even if patients were unable or unwilling to go in person. The clinicians with the largest relative increases in the number of encounters were hospitalists (7.11%), APRNs and PAs (6.60%), and other practitioners (4.00%). HSAs with medium telehealth intensity saw no significant change in clinician encounters, except for increasing visits with APRNs and PAs. These findings suggest that telehealth maintained or increased access to care, but differentially by specialty. The relative increase in hospitalist visit rates in high telehealth areas may reflect the growth of this specialty.^23^ The other practitioners category includes clinicians such as psychologists, social workers, physical therapists, and podiatrists. Increased encounters with clinicians in this category in high telehealth areas likely relates to telehealth-associated mental health care access, especially in urban areas.^24^

Consistent with increased clinician encounters, the high telehealth HSAs saw statistically significant increases in physician services cost. These HSAs also had statistically significant increases in SNF, DME, and inpatient care cost and statistically significant decreases in home health and outpatient care cost. Although increasing costs in more intensive inpatient care during the height of the pandemic makes sense, in medium HSAs, only DME costs increased significantly. The observed increase in inpatient care cost in high HSAs is consistent with the relative increase in ACS hospitalizations in those areas and may be another sign of higher capacity in these areas.

### Limitations

This study has limitations that should be mentioned. The use of data from the second half of 2021 prevents the measurement of postpandemic outcomes. This period overlapped with the surge in COVID-19 cases due to the Delta variant, which peaked in September 2021, and the beginning of the surge due to the Omicron variant. Therefore, we cannot determine the associations of telehealth with quality, access, and cost independently of the PHE. In particular, the finding that telehealth intensity was associated with more hospitalizations but not ED visits is likely affected by the PHE. For example, if a patient delayed care because of concerns about COVID-19 but then had a telehealth encounter because of an acute concern, they may have then sought and been admitted for inpatient care rather than further delaying care, adding to the ACS hospitalizations for the high telehealth intensity HSAs. The overall pattern during a period when there were fewer concerns and capacity limits to in-person care could look very different.

## Conclusions

In this cohort study of Medicare beneficiaries within 3436 HSAs near the end of the COVID-19 public health emergency in the US, HSAs with the most telehealth usage improved access to care (as measured by clinician encounters at the HSA level) and attenuated the decline in ACS hospitalizations compared with other areas. Additional analysis using data from after the PHE and with alternative outcome measures could help produce estimates that, being subject to fewer confounding factors, better capture the potential beneficial effects of telehealth on the quality of care.

## Notes

Supplement 1. **eAppendix 1.** Robustness Checks **eAppendix 2.** Effect of Separating Rural and Urban HSAs on the Impact Estimates **eAppendix 3.** Parallel Trends Assumption

Supplement 2. **eTable 1.** Impact Estimates for Risk-Adjusted ACS per 1,000 Beneficiaries; Hospitalizations **eTable 2.** Impact Estimates for Risk-Adjusted ACS per 1,000 Beneficiaries; Hospitalizations (Propensity Score Weighted) **eTable 3.** Impact Estimates for Risk-Adjusted ACS per 1,000 Beneficiaries; ED Visits **eTable 4.** Impact Estimates for Risk-Adjusted ACS per 1,000 Beneficiaries; ED Visits (Propensity Score Weighted) **eTable 5.** Impact Estimates for Clinician Encounters per Beneficiary **eTable 6.** Impact Estimates for Clinician Encounters per Beneficiary (Propensity Score Weighted) **eTable 7.** Impact Estimates for Total Cost of Care per Beneficiary **eTable 8.** Impact Estimates for Total Cost of Care per Beneficiary (Propensity Score Weighted) **eTable 9.** Impact Estimates Using Quartiles of Telehealth Intensity **eTable 10.** Propensity-Score Weighted Impact Estimates Using Quartiles of Telehealth Intensity **eTable 11.** Impact Estimates Omitting Small HSAs **eTable 12.** Propensity-Score Weighted Impact Estimates Excluding Small HSAs **eTable 13.** Parallel Trends Assumption **eTable 14.** Sample Characteristics (Means) by Telehealth Tercile and Year

Supplement 3.
Data Sharing Statement
