# Supplementary material for: Telehealth Expansion and Medicare Beneficiaries’ Care Quality and Access
Source: JAMA Netw Open. 2024 May 13;7(5):e2411006. doi: 10.1001/jamanetworkopen.2024.11006 (PMC11091757; doi:10.1001/jamanetworkopen.2024.11006)
Supplement: Supplement 1. — eAppendix 1. Robustness Checks eAppendix 2. Effect of Separating Rural and Urban HSAs on the Impact Estimates eAppendix 3. Parallel Trends Assumption [file jamanetwopen-e2411006-s001.pdf]

Supplemental Online Content

Saharkhiz M, Rao T, Parker Lue, Borelli S, Johnson K, Cataife G. Telehealth expansion and Medicare beneficiaries' care quality and access. *JAMA Netw Open*. 2024;7(5):e2411006. doi:10.1001/jamanetworkopen.2024.11006

eAppendix 1. Robustness Checks
eAppendix 2. Effect of Separating Rural and Urban HSAs on the Impact Estimates
eAppendix 3. Parallel Trends Assumption

This supplemental material has been provided by the authors to give readers additional information about their work.

© 2024 Saharkhiz M et al. *JAMA Network Open.*

eAppendix 1: Robustness Checks

Supplemental Tables 1-12 present robustness checks for the results presented in this research:

1. Geographic Adjustment: eTables 1-8, Full Controls + GAFs Model
2. Utilization: eTables 1-8, Full Controls + Utilization Model
3. Propensity Score Weighting: eTables 2, 4, 6, 8, 10, 12 all models
4. Sensitivity to Outliers: eTables 9 and 10
5. Omitting Small HSAs: eTables 11 and 12

1. Controlling for Geographic Adjustment Factors

Medicare payments change from year to year because of changes in the geographic adjustment factors (GAFs). Because the GAFs vary across HSAs and because they enter the payments multiplicatively (as opposed to additively), a DID strategy cannot perfectly adjust for them, and it is better to make the cost comparable across HSAs before estimating the model. CMS has developed standardized payment amounts for this purpose. Standardized payment amounts are hypothetical Medicare payments calculated as if claims were priced based on the national amounts without adjusting for GAFs or including other factors that make a cross-sectional comparison invalid, such as payments for indirect medical education (CMS, 2020). However, because of lack of access to complete data on standardized payment amounts, we used the actual paid amount. Therefore, it is possible that results are driven by the differences in the GAFs as opposed to differences in resource use between areas of varying telehealth intensities.

As a robustness check, we constructed a measure of the hospital wage index from the Inpatient Prospective Payment System (IPPS) and measures of geographic practice cost indexes (physician work, practice expense, and malpractice insurance, abbreviated GPCI) from the PFS by HSA and semester. We included these four measures as covariates in the DID regressions for all outcomes, but their inclusion did not affect the findings qualitatively; there were only modest changes in impact estimates. For example, after controlling for GAFs, the estimate for the impact of High telehealth intensity on the total cost of care, relative to Low telehealth intensity, decreased only by $4.25 per beneficiary per semester, from $164.99 to $160.74, which is a negligible amount. eTables 1-8 (Full Controls + GAFs model) present the impact estimates after adjusting for the differences in GAFs for the main outcomes.

2. Utilization

Our main specification does not control for in-person utilization. This is because controlling for the changes in in-person utilization would have prevented us from capturing the effects that telehealth use may have on outcomes through the follow-up in-person visits. Indeed, more telehealth use may lead to more in-person use via downstream effects, which in turn may cause better outcomes. However, not controlling for in-person utilization could cause an omitted variable bias in our findings because the effect of telehealth usage may be confounded with the effect of general health care utilization. Therefore, as a robustness check, we controlled for in-person utilization in a separate analysis.

We constructed a measure of in-person utilization as the number of non-telehealth visits per FFS Medicare beneficiary by HSA and semester and included it as a covariate in the DID regressions for quality and cost outcomes. A comparison of the various models (eTables 1-8; Full Controls + Utilization model) shows that the impact estimates do not change substantially after adjusting for the differences in in-person utilization, reducing concerns that the findings are driven by the omitted variable bias.

3. Propensity Score Weighting

A DID methodology is robust to differences that do not change over time and accounts for differences that do change over time through the covariates included in the model. However, because the three telehealth intensity groups are different in many observed characteristics, they may also be different in unobserved characteristics. For example, at the time of this study, market characteristics data were unavailable for 2021, and, as a result, we could not include them in the model. Propensity score weighting (PSW) can reduce these concerns by making Low, Medium, and High groups more comparable on observed characteristics; presumably, when the groups are similar on observed characteristics, they are similar on unobserved characteristics as well.

To this aim, we used multinomial logistic regression to estimate the propensity scores for each HSA (i.e., the probability of that HSA having Low, Medium, or High telehealth intensity) based on their observed characteristics during the second semester of 2019. Then, we weighted each HSA in the DID regressions by the inverse of their propensity score for their observed telehealth intensity; this resulted in propensity score-weighted DID impacts. Statistically speaking, these estimates are calculated such that HSAs that are similar to HSAs in their respective group will be weighted less, while HSAs that are similar to HSAs in other groups will be weighted more. This increases comparability and reduces the potential impact of confounders on the results.

Changes in the results can be interpreted as differences that occur because HSAs that are less likely to be included in a given tercile are weighted more. For instance, the average total cost of care was positive and significant in the main result; after propensity score weighting, it is still positive and significant, but the magnitude of the effect is larger. This indicates that the total cost of care increased more in High telehealth intensity HSAs that were less likely to be included in that group.

We can see in Table 2 that there were differences between Low, Medium, and High telehealth intensity groups for many characteristics. After applying the propensity score weights, the differences were statistically insignificant for the majority of characteristics. After applying the propensity score weights to the DID regressions, the impact estimates did not change qualitatively for the main outcomes with the exception of ACS ED visits. Investigating this further, we found that the increase in ED visits disappeared if we excluded the small HSAs (eTable 12). In addition, we know from MedPAC's prior work that the ACS ED visits measure is unreliable when HSAs with a small number of beneficiaries are included in the analysis. Thus, propensity score weighted DID estimates concur with the main findings, which increases our confidence in them.

© 2024 Saharkhiz M et al. *JAMA Network Open.*

4. Sensitivity to Outliers

To check the sensitivity of these results to outliers, we replicated the analyses in Table 3 for our main outcomes, but using quartiles instead of terciles. These results largely replicated our results, with statistically significant results for the highest quartiles of telehealth intensity.

To investigate the possibility that the results are being influenced by extreme values in the outcomes, we have plotted the distributions of the main outcome variables by telehealth treatment group. The distribution of values is similar or slightly compressed for the High telehealth group, which suggests that our findings are not caused by outlier values of the outcome variable.

Exhibit 1-1: Distribution of ACS Hospitalizations by Telehealth Intensity, 2021-Semester 2

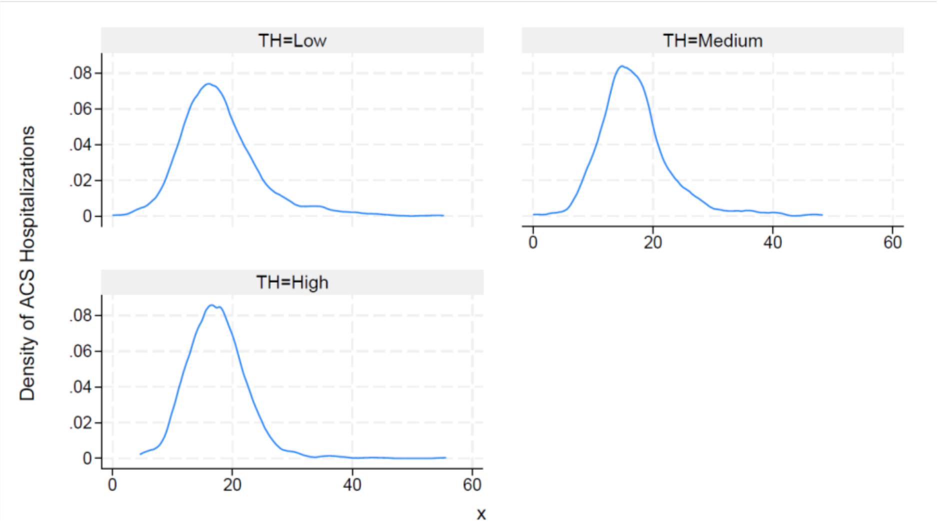

Exhibit 1-2: Distribution of ACS ED Visits by Telehealth Intensity, 2021-Semester 2

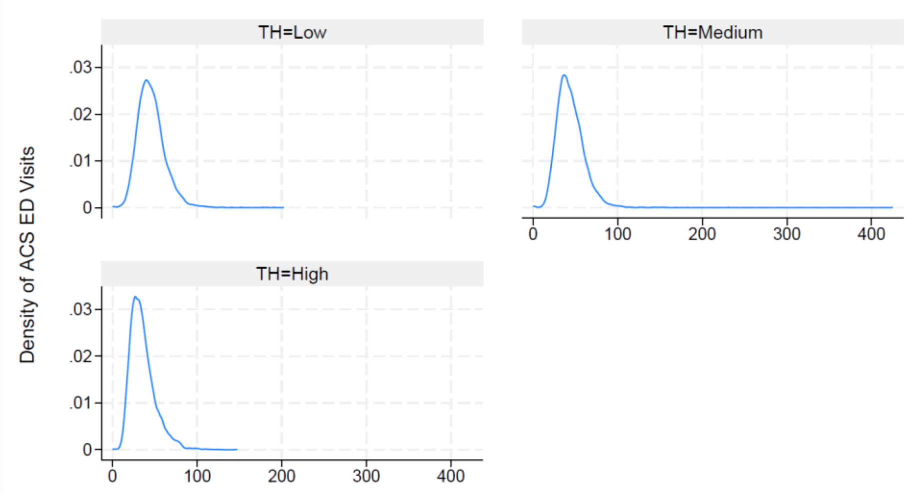

© 2024 Saharkhiz M et al. *JAMA Network Open.*

Exhibit 1-3: Distribution of Average Clinician Encounters per Beneficiary by Telehealth Intensity, 2021-Semester 2

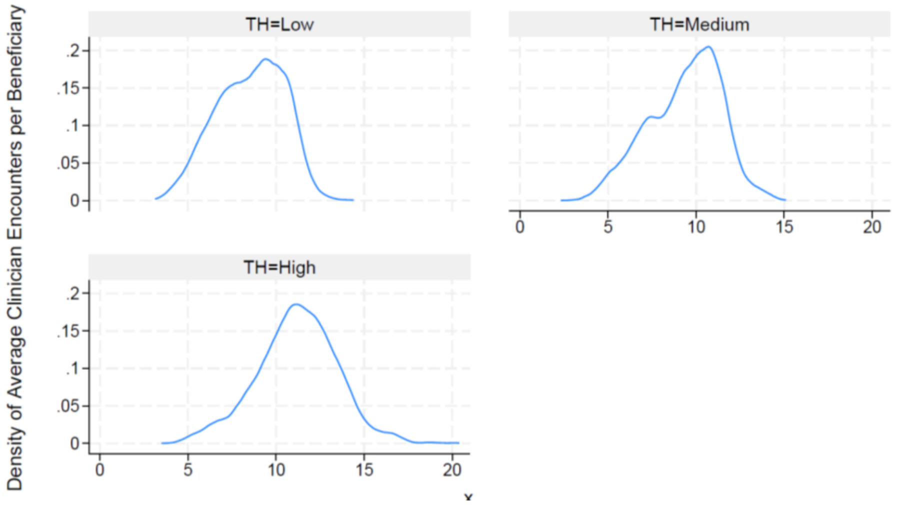

Exhibit 1-4: Distribution of Average Total Cost of Care per Beneficiary by Telehealth Intensity, 2021-Semester 2

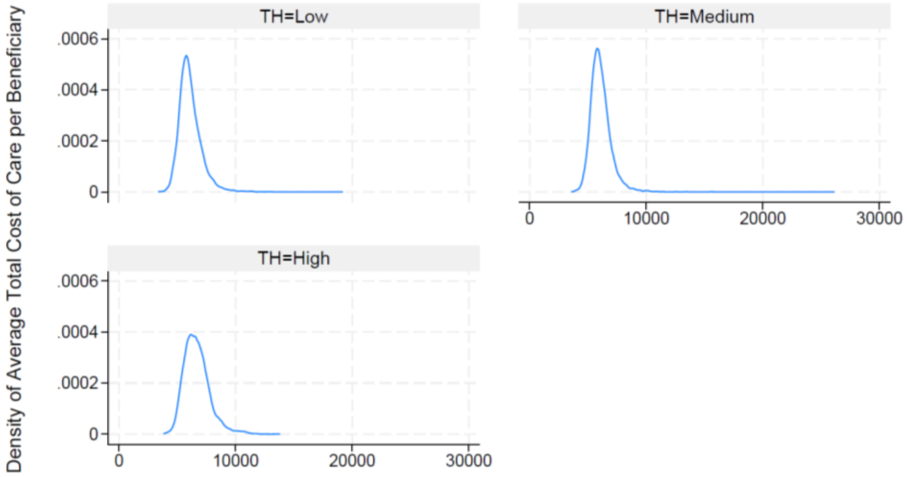

5. Omitting Small HSAs

MedPAC's prior work on ACS hospitalizations and ED visit rates has shown that measures calculated using a denominator with fewer than 500 beneficiaries have low reliability. Thus, as a robustness check, we excluded small HSAs from the sample and repeated the analysis. Small HSAs are defined as HSAs that have fewer than 500 FFS beneficiaries during the study period. This definition resulted in the exclusion of 162 HSAs out of 3,436 HSAs that are in the main sample. eTables 11 and 12 present the findings for the main outcomes. As the exhibit shows, excluding small HSAs has a negligible effect on the impact estimates.

eAppendix 2: Effect of Separating Rural and Urban HSAs on the Impact Estimates

1. Background

One of the biggest differences between the different telehealth intensity groups is level of urbanicity. As noted in the report, the average percentage of beneficiaries living in an urban area was 24, 41, and 77 for the Low, Medium, and High groups, respectively. Given the higher urbanicity of the High telehealth intensity group, one may be concerned that the differences in outcomes are due to differences in urbanicity levels, rather than due to differences in telehealth usage.

Exhibit 2-1: High Telehealth HSAs are More Likely to be in Urban Areas

HSAs by tercile of telehealth services per 1,000 beneficiaries

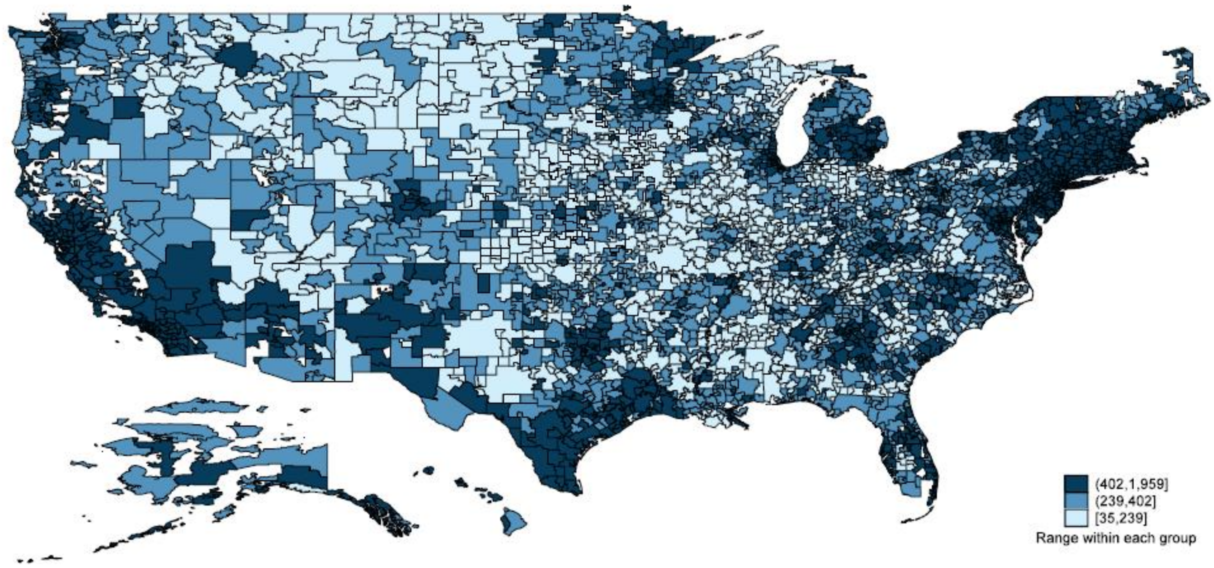

As a robustness check, we separated HSAs into rural and urban subsamples and repeated the DID analysis using them. That is, we calculated the impact of High and Medium telehealth intensity relative to Low telehealth intensity for urban and rural areas separately. If the results in the report are a consequence of urbanicity (and not because of telehealth usage) then we would expect that splitting the sample would cause the results to disappear—e.g., among urban HSAs, increasing or decreasing telehealth usage should have no impact on our outcome measures.

2. Splitting the Sample

We split the sample by the median share of beneficiaries living in urban areas in the second semester of 2019 (15.7%). HSAs above the median were classified as urban, and HSAs below the median were classified as rural. Choosing other values, e.g., the mean (47.4%), as

© 2024 Saharkhiz M et al. *JAMA Network Open.*

the threshold for urbanicity does not affect the analysis because beneficiaries in most HSAs live in the same type of area (Exhibit 2). We chose the median to generate two equal-sized subsamples.

Exhibit 2-2: Most HSAs are Fully Urban or Rural

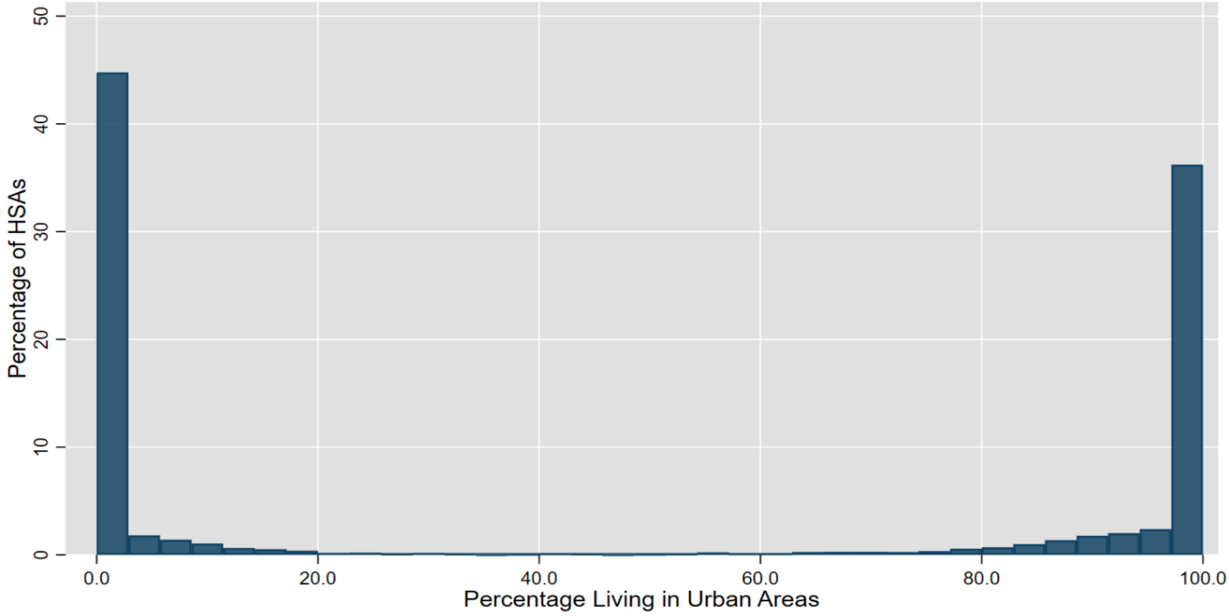

Notes. Data is from the second semester of 2019.

Exhibit 3 presents a crosstabulation of HSAs by telehealth intensity and urbanicity. Most HSAs in the urban subsample are in High telehealth intensity group while most HSAs in the rural subsample are in the Low telehealth intensity group. Due to the smaller sample sizes of subsamples, the impact estimates will become less precise, particularly for the High group in the rural subsample.

Exhibit 2-3: Crosstabulation of HSAs by Telehealth Intensity and Urbanicity

	Urban	Rural
Low	305	829
Medium	514	655
High	899	234
**Total**	1718	1718

3. Results

Exhibit 4 presents a comparison of the impact estimates for the main outcomes based on the full sample and the urban and rural subsamples. Overall, the impact estimates follow the same general pattern as the full sample, which shows that telehealth intensity has a similar impact even when we restrict our analysis to urban or rural HSAs. This suggests that the association between telehealth intensity and outcomes is not caused by different levels of urbanicity between the Low, Medium, and High groups. We provide more details below.

© 2024 Saharkhiz M et al. *JAMA Network Open.*

Exhibit 2-4: Effect of Splitting the Sample on the Impact Estimates

Outcome	Full Sample	Urban Sample	Rural Sample
Panel A: **Medium** Telehealth Intensity (relative to Low)			
ACS hospitalizations per 1,000 beneficiaries per semester	0.41 (1.70%)	0.26 (1.12%)	0.27 (1.07%)
ACS ED visits per 1,000 beneficiaries per semester	-0.58 (-1.29%)	-0.68 (-1.74%)	-0.69 (-1.38%)
Clinician encounters per beneficiary per semester	0.02 (0.22%)	0.03 (0.31%)	0.04 (0.46%)
Total cost of care per beneficiary per semester (in dollars)	18.08 (0.30%)	57.91 (0.96%)	15.47 (0.25%)
Panel B: **High** Telehealth Intensity (relative to Low)			
ACS hospitalizations per 1,000 beneficiaries per semester	1.63 (6.90%) ***	1.30 (5.61%) ***	0.92 (3.65%)
ACS ED visits per 1,000 beneficiaries per semester	0.10 (0.27%)	-0.08 (-0.25%)	-1.00 (-2.02%)
Clinician encounters per beneficiary per semester	0.30 (2.67%) ***	0.36 (3.06%) ***	0.19 (2.09%) **
Total cost of care per beneficiary per semester (in dollars)	164.99 (2.47%) ***	211.69 (3.13%) ***	2.31 (0.04%)

Notes. ACS = ambulatory care sensitive. The denominator for the percentages is the baseline average. ***, **, and * denote statistical significance at 1, 5, and 10% level, respectively.

Medium Telehealth Intensity: As in the full sample, there are no statistically significant differences in the main outcomes between the Medium and the Low groups based on the rural or urban subsamples (Exhibit 4, Panel A).

High Telehealth Intensity: The impact estimates for the High group based on the urban subsample show the same patterns as in the full sample (Exhibit 4, Panel B). However, the impact estimates based on the rural subsample show no effect on the total cost of care and are smaller than the urban subsample for the other main outcomes (Exhibit 4, Panel B). For ACS hospitalizations, the result is no longer significant for the rural subsample, but this is likely due to a less precise estimate; we cannot say whether this impact estimate is statistically significantly different from the impact in the urban subsample. In addition, while the High group in the rural subsample does not show an effect on the total cost of care for all claim types combined, the total cost of care for physician services shows an increase, consistent with the increase in clinician encounters per beneficiary (Exhibit 5).

Exhibit 2-5: Effect of Splitting the Sample on the Total Cost of Care per Beneficiary per Semester (in Dollars)

	Full Sample	Urban Sample	Rural Sample
All Claim Types	164.99 (2.47%) ***	211.69 (3.13%) ***	2.31 (0.04%)
Inpatient	63.63 (3.22%) ***	88.41 (4.38%) ***	-23.91 (-1.33%)

© 2024 Saharkhiz M et al. *JAMA Network Open.*

	Full Sample	Urban Sample	Rural Sample
Outpatient	-31.93 (-1.98%) *	6.73 (0.44%)	-31.85 (-1.60%)
Skilled Nursing Facility	45.14 (9.51%) **	21.70 (4.63%) *	19.07 (3.82%)
Home Health	-20.47 (-7.60%) ***	-17.89 (-6.34%) ***	-6.59 (-3.00%)
Hospice	-1.60 (-1.27%)	4.12 (3.13%)	-6.54 (-6.15%)
Physician	100.54 (4.89%) ***	97.44 (4.46%) ***	48.84 (3.14%) **
Durable Medical Equipment	9.68 (6.12%) ***	11.17 (7.32%) **	3.29 (1.83%)

Notes. Impact estimates are for the High telehealth intensity group and they are relative to the Low telehealth intensity. The denominator for the percentages is the baseline average in the relevant sample or subsample. ***, **, and * denote statistical significance at 1, 5, and 10% level, respectively.

4. Discussion

As described above, we found that the effects tend to be smaller for rural HSAs than for urban HSAs for all the main outcomes. A potential explanation for this observation is that most of the rural HSAs in the High group had telehealth intensity levels just above the threshold, with relatively few HSAs at higher levels of telehealth usage. In contrast, urban HSAs in the High group are less concentrated at the threshold, with many HSAs at much higher levels of telehealth intensity (Exhibit 5). Thus, it is possible that the impact estimates for the High group based on the urban subsample are larger than rural subsample because telehealth usage is higher in urban areas. This in turn could be due to various reasons, such as urban areas having been affected by COVID-19 pandemic differently, or urban areas having reacted to the pandemic differently in terms of their capacity and velocity to restore healthcare services.

© 2024 Saharkhiz M et al. *JAMA Network Open.*

Exhibit 2-6: Telehealth Intensity is Higher in Urban HSAs of the High Group

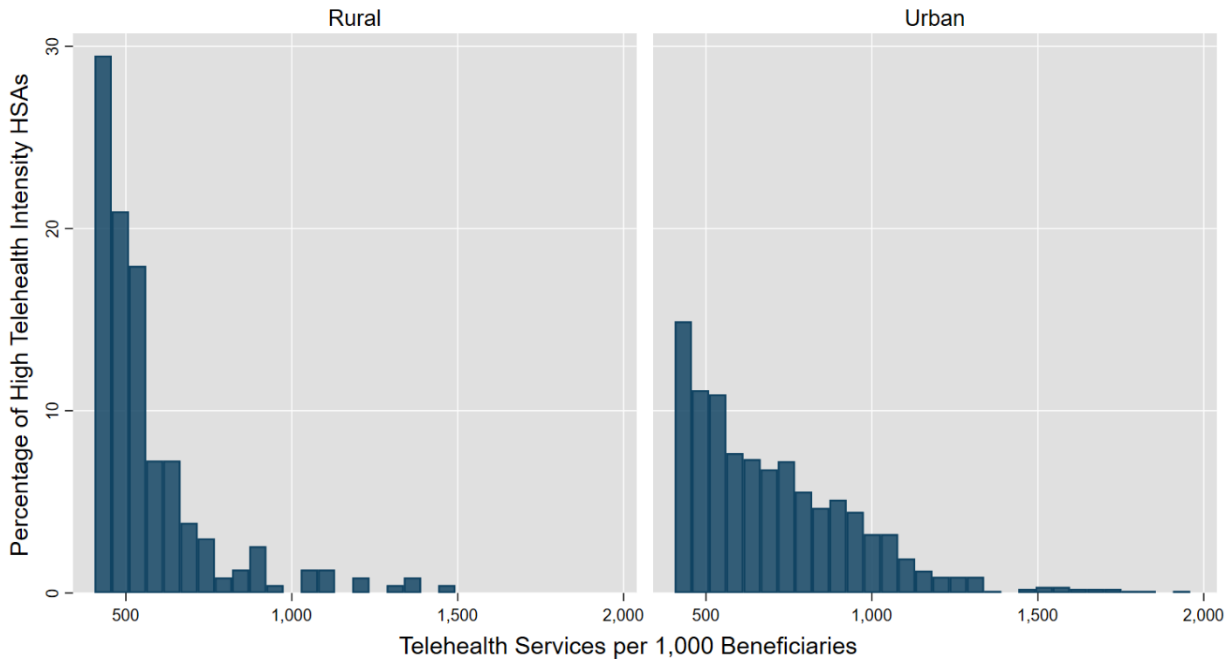

5. Conclusions

We found that limiting the sample to only urban or rural HSAs does not eliminate the association between High telehealth intensity and outcomes. This indicates that the relative urbanicity of the High group is not the cause of the association between telehealth intensity and outcomes. We do find variations in the findings between the urban and rural subsamples, primarily that the association between telehealth intensity and outcomes are smaller in rural areas relative to urban areas. While this could be a potential avenue for future research, it does not provide evidence to contradict our findings.

© 2024 Saharkhiz M et al. *JAMA Network Open.*

eAppendix 3: Parallel Trends Assumption

In general, the DID estimates capture associations between telehealth intensity and the outcomes. A necessary condition to interpret these associations as causal (i.e., that the difference in outcomes between the Low, Medium, and High telehealth intensity groups is due to the difference in the usage of telehealth services) is that the Low telehealth intensity group provides a valid counterfactual for the outcomes in the Medium or High groups in the absence of telehealth expansion. This assumption is not testable, but we have more confidence in its validity if the outcomes for the Low, Medium, and High telehealth intensity HSAs moved in parallel (i.e., had similar patterns) before the expansion of telehealth. This is usually referred to as the parallel trends assumption test.

We checked whether there is a statistically significant difference in outcomes between the Low and Medium groups and between the Low and High groups in 2018 and 2019 after adjusting for the differences in the covariates following the same procedure described in the paper (eTable 13). We found that for Medium telehealth intensity HSAs, the parallel trends test passes for three of the four main outcomes (ACS hospitalizations, ACS ED visits, and total cost of care) and fails for the clinician encounters outcome. In addition, for High telehealth intensity HSAs, the parallel trends test passes for two of the four main outcomes (ACS ED visits and total cost of care) and fails for the other main outcomes (ACS hospitalizations and clinician encounters). However, a visual inspection of the trends during the same period shows that the trends are largely similar (see Figure 1, reproduced below):

Figure 1: Trends in Outcome Variables

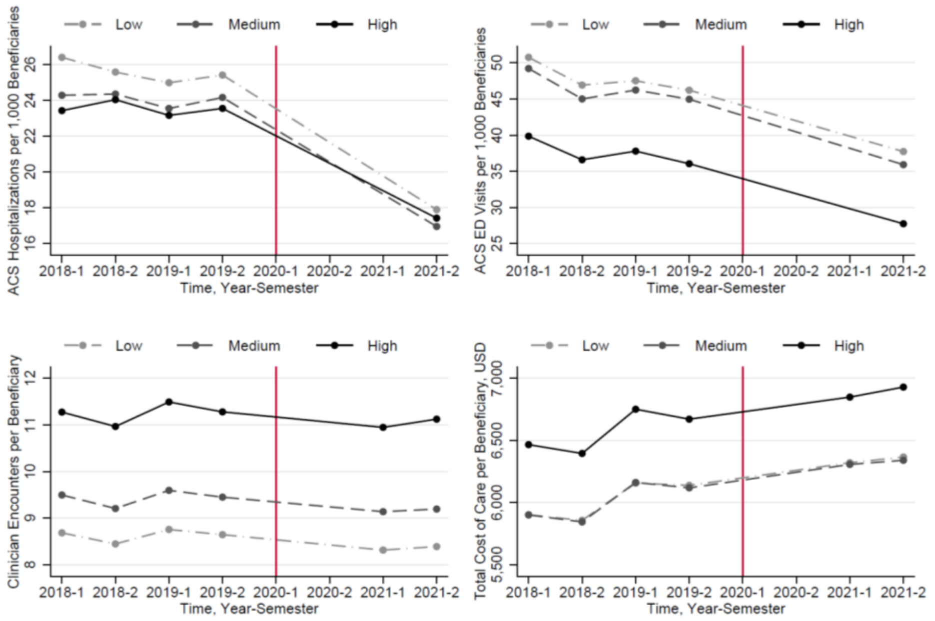

Note: the red line shows the beginning of the Public Health Emergency (PHE).

© 2024 Saharkhiz M et al. *JAMA Network Open.*

For instance, looking at a graph of the trends for clinician encounters, we see that HSAs in the Low, Medium, or High groups did have very similar trends before the expansion of telehealth even though the trends were statistically different (Figure 1). Looking at the magnitude of the difference, although the difference in the pre-period is statistically significantly different from zero, the post-period differences are nearly 3 times greater and statistically significantly different from the pre-period.

Thus, we conclude that the violations of the parallel trends assumption for some outcome/semesters detected by the formal tests are primarily driven by the high precision of our estimates. The small magnitude of the differences from zero suggests that these violations are not a major concern.

© 2024 Saharkhiz M et al. *JAMA Network Open.*
