# Supplementary material for: Telehealth Expansion and Medicare Beneficiaries’ Care Quality and Access
Source: JAMA Netw Open. 2024 May 13;7(5):e2411006. doi: 10.1001/jamanetworkopen.2024.11006 (PMC11091757; doi:10.1001/jamanetworkopen.2024.11006)
Supplement: Supplement 3. — Data Sharing Statement [file jamanetwopen-e2411006-s003.pdf]

Data Sharing Statement

Saharkhiz. Telehealth Expansion and Medicare Beneficiaries' Care Quality and Access. *JAMA Netw Open*. Published May 13, 2024. doi:10.1001/jamanetworkopen.2024.11006

Data
Data available: No

Additional Information
Explanation for why data not available: The data used in this study are based on protected health records of Medicare beneficiaries, and we do not have authorization from Medicare to share these data.
